# Thermal Conductivity of 3C/4H-SiC Nanowires by Molecular Dynamics Simulation

**DOI:** 10.3390/nano13152196

**Published:** 2023-07-28

**Authors:** Kaili Yin, Liping Shi, Xiaoliang Ma, Yesheng Zhong, Mingwei Li, Xiaodong He

**Affiliations:** 1 Center for Composite Materials and Structures, Harbin Institute of Technology, Harbin 150080, China; 2 School of Material Science and Engineering, Harbin Institute of Technology, Harbin 150001, China; 3 Shenzhen STRONG Advanced Materials Research Institute Co., Ltd., Shenzhen 518000, China

**Keywords:** SiC, stacking faults, thermal conductivity, molecular dynamics simulation

## Abstract

Silicon carbide (SiC) is a promising material for thermoelectric power generation. The characterization of thermal transport properties is essential to understanding their applications in thermoelectric devices. The existence of stacking faults, which originate from the “wrong” stacking sequences of Si–C bilayers, is a general feature of SiC. However, the effects of stacking faults on the thermal properties of SiC are not well understood. In this study, we evaluated the accuracy of Tersoff, MEAM, and GW potentials in describing the thermal transport of SiC. Additionally, the thermal conductivity of 3C/4H-SiC nanowires was investigated using non-equilibrium molecular dynamics simulations (NEMD). Our results show that thermal conductivity exhibits an increase and then saturation as the total lengths of the 3C/4H-SiC nanowires vary from 23.9 nm to 95.6 nm, showing the size effect of molecular dynamics simulations of the thermal conductivity. There is a minimum thermal conductivity, as a function of uniform period length, of the 3C/4H-SiC nanowires. However, the thermal conductivities of nanowires weakly depend on the gradient period lengths and the ratio of 3C/4H. Additionally, the thermal conductivity of 3C/4H-SiC nanowires decreases continuously from compressive strain to tensile strain. The reduction in thermal conductivity suggests that 3C/4H-SiC nanowires have potential applications in advanced thermoelectric devices. Our study provides insights into the thermal transport properties of SiC nanowires and can guide the development of SiC-based thermoelectric materials.

## 1. Introduction

Silicon carbide (SiC) exhibits excellent physical and chemical properties, including excellent mechanical properties, thermochemical stability, and wide bandgap energy, which is attracting much attention for its thermoelectric properties [1]. SiC exhibits over 200 polytypes, which result from different periodic stacking sequences of Si–C bilayers along the *c* axis. Stacking faults are common in SiC and arise from “wrong” stacking sequences. The experimentally determined formation energies of stacking faults are 3~15 mJ/m^2^ (e.g., 2.9 ± 0.6 mJ/m^2^ and 14.7 ± 2.5 mJ/m^2^ for 6H and 4H-SiC single crystals, respectively) [2], about an order of magnitude lower than the energies of the corresponding defects in cubic semiconductors (45 mJ/m^2^, 55 mJ/m^2^, and 279 ± 41 mJ/m^2^ for GaAs, Si, and diamond, respectively) [3,4,5], leading to variations in the local stacking sequence in any of the crystalline forms [6].

Many methods, such as spark plasma sintering (SPS), physical vapor transport (PVT), chemical vapor deposition (CVD), etc., have been applied to fabricated SiC (Table 1). During the fabrication process, stacking faults can arise from the “wrong” stacking sequences of Si–C bilayers. According to the report, the triangular defects, which were intrinsically 3C-SiC polytype inclusions, were observed in the fabrication of 4H-SiC epitaxial layers grown via the CVD method [7,8]. These triangular defects showed different surface morphologies [9,10,11,12,13,14]. Studies on the 4H/3C boundary have shown that the 4H and 3C structures exhibit a coherent crystallographic orientation relationship, and the (0001) planes in the 4H-SiC are coherently connected to the (111) planes of the 3C-SiC [9]. Additionally, 3C-SiC nanowires with different states of stacking faults were observed. Zhijiang Wang et al. fabricated 3C-SiC nanowires with different states of stacking faults by varying the heating temperature [15]. Je-Hyung Kim et al. found that CVD-grown 3C-SiC nanowires may include hexagonal inclusions that are a few nanometers thick (4H- or 6H-SiC) by stacking faults [16]. Apart from the stacking faults that may occur during the preparation process, phase separation of solid-state binary compounds (e.g., 4H-SiC) induced by laser–material interaction has been observed [17,18,19]. Insung Choi et al. found that the (111)-oriented 3C-SiC layer was epitaxially grown on (0001)-oriented 4H-SiC under a single pulse from the laser. Moreover, the 4H-SiC surface with 10 irradiation pulses showed a decreased thickness of the 3C-SiC layer [19]. As common “wrong” stacking sequences, stacking faults can enhance phonon scattering and reduce thermal conductivity [20]. However, only a limited number of papers have described the influence of stacking faults on the performance of the SiC [21,22,23,24,25].

To design high-performance and reliable SiC thermoelectric power generation devices, understanding the influence of stacking faults on thermal conductivity is inevitable. Molecular dynamics (MD) simulation is a powerful tool that has made great progress in predicting the thermal and mechanical properties of nanoscale materials. Among them, ab molecular dynamics simulation has been applied to investigate the structural, strain, as well as thermal properties of complex nanostructures [38,39]. Non-equilibrium molecular dynamics (NEMD) simulation has been widely used in interfacial thermal transport properties of nanoscale materials with millions or tens of millions of atoms [40,41]. In the NEMD simulations, the accuracy of the results depends on the appropriate interatomic potentials. Many kinds of empirical potentials have been used to achieve the thermal conductivity of SiC [42,43,44]. Nevertheless, the applicability of the empirical interatomic potentials for calculating the thermal conductivity of SiC has not been verified from the perspective of phonon transport.

In this paper, the thermal conductivity of SiC containing stacking faults was investigated using NEMD simulation. Firstly, we explore the accuracy of Tersoff, MEAM, and GW potentials in describing the thermal transport of SiC. Additionally, we systematically reveal the relationship between thermal conductivity and period length in the 3C/4H-SiC nanowires. We also investigate the influence of temperature and strain on the thermal conductivity of 3C/4H-SiC nanowires.

## 2. Computation Details and Methods

### 2.1. Crystal Structural and Modeling Details

**Figure 1:**
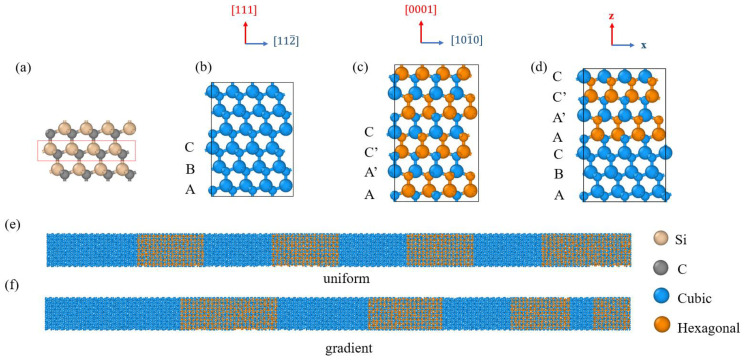
The structures of SiC: (**a**) fundamental bilayer; (**b**) 3C-SiC; (**c**) 4H-SiC; (**d**) 3C/4H-SiC; (**e**) uniform period length distribution; and (**f**) gradient period length distribution.

SiC polytypes are characterized by the periodical stacking sequence of the fundamental Si–C bilayer (Figure 1a) along the *c* axis. There are three possible arrangements of atoms in a fundamental bilayer of SiC structure, called the A, B, and C positions. Cubic polytype 3C-SiC shows the zincblende lattice (consistent with diamond) formed by the stacking sequence {ABCABC…} (Figure 1b). Hexagonal polytype 4H-SiC shows the stacking sequence {AA’C’CAA’C’C…}, where A’ denotes p-rotated A plane (Figure 1c). The arrangement of the next stacking fundamental bilayer of A can be B or A’ along the *c*-axis direction. The structure with the stacking sequence {…ABCAA’C’C…} is defined as the 3C/4H-SiC (Figure 1d). The Open Visualization Tool (OVITO) is applied to identify the diamond structure [45]. In this work, the thermal conductivity of 3C/4H-SiC nanowires with different period length distributions (uniform and gradient) was investigated. The period length of the uniform 3C/4H-SiC nanowires was defined as the total thickness of a pair of continuous 3C and 4H layers (Figure 1e). The period length of the gradient period 3C/4H-SiC nanowires was calculated as the sum of the average length of the 3C and 4H (Figure 1f) [46,47].

### 2.2. Molecular Dynamics Simulations

**Figure 2:**
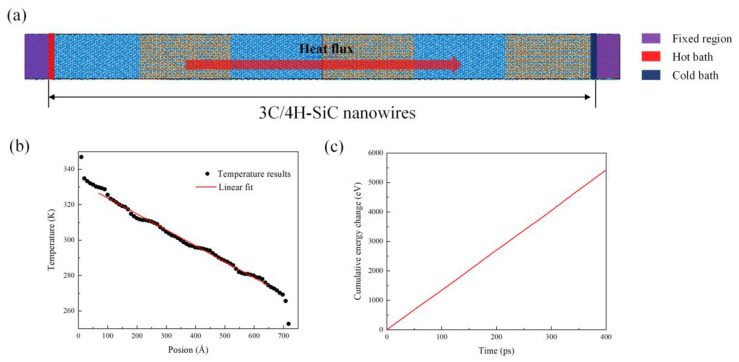
NEMD thermal conductivity calculations. (**a**) Schematic of NEMD simulation. (**b**) Temperature profiles along the heat flux direction. (**c**) Cumulative energy change as a function of time. The period and total lengths were 179.40 Å and 717.60 Å, respectively. Temperature profiles and cumulative energy change were obtained by Tersoff-1990 potential.

NEMD simulations were performed using the large-scale atomic/molecular massively parallel simulation package LAMMPS [48] to calculate the thermal conductivity of the 3C/4H-SiC nanowires. The schematic of the simulation setup is shown in Figure 2a. The dimensions of the model were 42 Å × 42 Å × n Å in the *x*, *y*, and *z* directions, where n ranged from 239 Å to 956 Å. Initially, an NPT ensemble (constant particle number, pressure, and temperature) using a Nosé–Hoover thermostat and barostat was conducted for 50 ps for the purposes of eliminating unreasonable stress at the interface and obtaining the optimized structure, after the system energy was minimized using the conjugate gradient algorithm under the periodic boundary conditions in all directions. In the second step, we set 3.0 nm long regions at both ends as “rigid walls”. A hot bath and a cold bath were placed at the left and right ends of the 3C/4H-SiC nanowires. The periodic boundary conditions were applied in the *x* and *y* directions, and the fixed boundary condition was used in the *z* direction. The heat flux was imposed along the *z* directions. The periodic boundary conditions used in the transverse directions (*x*, *y* directions) were to mimic the free surface of a free-standing nanowire [49]. Then, the system was maintained at equilibrium for 100 ps using the NVT ensemble (constant particle number, volume, and temperature). After the NVT, the system was run for 800 ps in an NVE ensemble (constant particle number, volume, and energy). To introduce non-equilibrium conditions, the hot and cold baths were maintained at ±50 K on the averaged temperature to create a temperature gradient in the 3C/4H-SiC region. A Langevin thermostat with a damping parameter of 0.1 ps was used to maintain the temperature of the 3C/4H-SiC region constant.

When the system reached a steady state, the heat flux $J_{z}$ and temperature gradient ∆T were obtained. The temperature gradient ∆T could be calculated by fitting the linear portion of the temperature profile in Figure 2b, and the heat flux $J_{z}$ could be calculated using the following equation:

(1) Jz=∆Q∆tA

where A is the cross-sectional area and ∆Q and ∆t are the energy difference and time interval of the simulation process, respectively. The thermal conductivity κ was calculated using Fourier’s law:

(2) κ=−Jz∂T∂z

where ∂T∂z is the temperature gradient along the *z* direction.

## 3. Results and Discussion

### 3.1. Empirical Potential Selection

**Figure 3:**
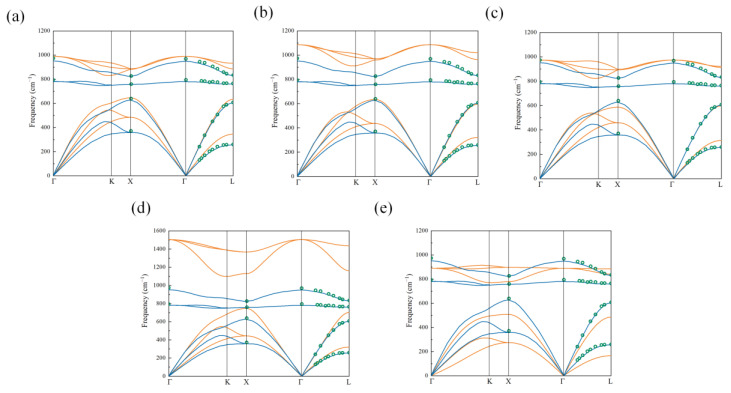
Phonon dispersion curves in 3C-SiC along the high-symmetry directions, obtained using several different potentials: (**a**) Tersoff-1989 [54]; (**b**)Tersoff-1990 [55]; (**c**) Tersoff–Erhart–Albe [56]; (**d**) MEAM95 [57]; and (**e**) GW02 [58]. Gold curves represent those from classical potentials. Green circles represent experimental data points [59] and blue curves represent DFT results [60] (reproduced with permission).

An accurate description of phonon dispersion could facilitate the investigation of thermal transport [50,51,52]. A 2 × 2 × 2 supercell was set up for 3C-SiC, and the phonon dispersion properties were obtained using LAMMPS [48] and the ALAMODE package [53]. Figure 3 shows the phonon dispersion curves in 3C-SiC along the high-symmetry directions obtained using Tersoff-1989 [54] (TR-89), Tersoff-1990 [55] (TR-90), Tersoff-Erhart-Albe [56] (TR-EA), MEAM95 [57], and GW02 [58] potentials. We also compared the results (gold curves in Figure 3) with the experimental [59] (green circles in Figure 3) and DFT values [60] (blue curves in Figure 3). In this paper, we only considered the effect of acoustic branch phonons on heat transport, because optical branch phonons were generally believed to contribute negligibly to heat transport [61]. As shown in Figure 3d, the acoustic branch phonons described using the MEAM95 potential were overestimated. As shown in Figure 3e, the GW02 potential provided lower acoustic branch frequencies. For Tersoff potentials, the TR-89, TR-90, and TR-EA potentials accurately described the acoustic branch phonons to a certain extent in the first Brillouin zone, as shown in Figure 3a–c. Compared with the TR-89 potential, the TR-90 and TR-EA potentials were able to more accurately describe the TA branch along the high-symmetry points Γ to L. At the L point, the experimentally measured frequency of the TA branch was 607.84 cm^−1^, while the TR-89, TR-90, and TR-EA potentials showed values of 633.09 cm^−1^, 595.7 cm^−1^, and 597.39 cm^−1^, respectively. Additionally, the frequency of the TA branch (634.23 cm^−1^) at the X point simulated by TR-90 potential fit well with the experimentally measured frequency (639.28 cm^−1^). Thus, the TR-90 potential is mostly suited to predict the heat transport of SiC, despite the higher optical phonon branches. From here on, TR-90 potential was used to investigate the thermal conductivity of SiC nanowires in the rest of the paper.

### 3.2. Effect of the Sample’s Total Length

**Figure 4:**
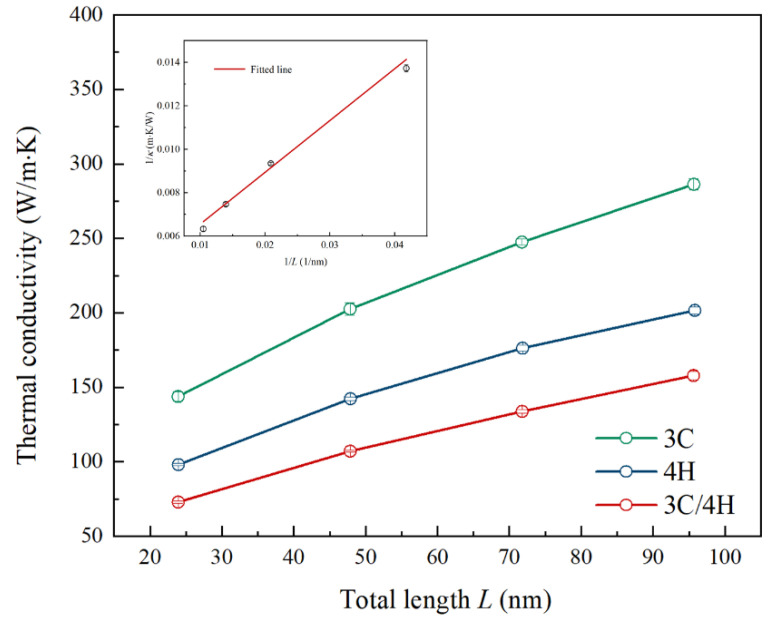
The thermal conductivity versus the SiC nanowire’s total length. The period length of the 3C/4H-SiC nanowires was fixed at 239.20 Å. The inset represents the reciprocal of *κ* as a function of the reciprocal of the 3C/4H-SiC lengths. The red line represents the linear fitted line, and the hollow circles represent the MD simulation data.

Figure 4 illustrates the thermal conductivity variations of the 3C, 4H, and 3C/4H-SiC nanowires at 300 K with different total lengths. The uniform period length of the 3C/4H-SiC nanowires was fixed at 239.14 Å. The results show an initial increase in thermal conductivity with increasing total length, followed by saturation as the total length further increased. This typical phenomenon originates from the size effect of the molecular dynamics simulation of the thermal conductivity [62]. At a finite simulation size, the system lacks long-wavelength phonons, and the maximum mean free path for all phonons is limited by the dimension of the simulation box. As shown in the insert of Figure 4, the reciprocal of the thermal conductivity (1κ) was linearly related to the reciprocal of the thermal conductivity (1L) for the uniform period length 3C/4H-SiC nanowires. Additionally, despite the atomically perfect interface and strong covalent bonds between 3C and 4H-SiC, there was still finite interfacial thermal resistance due to acoustic mismatch. This was evident from the finite temperature difference which can be observed at the interface in Figure 2b. We found that the thermal conductivity of the 3C/4H-SiC nanowires was significantly lower than that of the perfect monocrystalline 3C and 4H SiC at all simulation lengths. For instance, when the simulation length was about 95 nm, the thermal conductivity values of the 3C, 4H, and 3C/4H-SiC were 286.31 ± 3.93 W/m·K, 201.65 ± 2.41 W/m·K, and 157.86 ± 2.67 W/m·K, respectively.

### 3.3. Effect of Period Length

**Figure 5:**
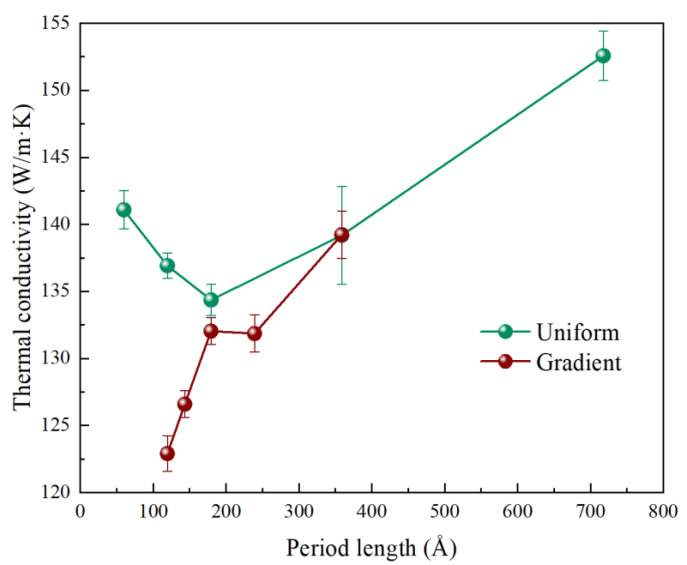
The dependence of thermal conductivity on the period length for the uniform and gradient 3C/4H-SiC nanowires at 300 K.

The effects of period length on thermal conductivity with a fixed total length of 717 Å were investigated. Figure 5 shows the relationship between thermal conductivity and period length. For the 3C/4H-SiC nanowires with uniform periods, the thermal conductivity exhibited nonmonotonic behavior with the increase in the period length. When the period length was 59.64 Å, 3C/4H-SiC nanowires showed a thermal conductivity of 141.08 ± 1.41 W/m·K. When the period length increased from 59.64 Å to 179.40 Å, the thermal conductivity gradually decreased and reached the minimum value of 134.36 ± 1.16 W/m·K. Then, with the increase in the period length, the thermal conductivity increased. The thermal conductivity was 139.19 ± 3.66 W/m·K with a period length of 358.73 Å. The existence of minimum thermal conductivity as a function of the period length of the superlattices has been observed in experiments and simulations [47,63,64]; it is an important characteristic of the coherent-to-incoherent transition. When the period length is below 179.40 Å, the formation of the minibands reduces the phonon group velocity. To the right of the minimum of thermal conductivity, the large period lengths provide enough distance for intrinsic phonon–phonon scatterings, weakening the coherent phonon modes over multiple periods. In this situation, the heat transfer is dominated by the incoherent phonons in the 3C/4H-SiC nanowires. With increasing period lengths, the phonon interface scattering weakens, thus enhancing the phonon mean free path. As a result, the thermal conductivity bounces back with a period length of 179.40 Å. For the 3C/4H-SiC nanowires with gradient period lengths, these structures remove the periodicity of the entire structure, thus disrupting the coherence of phonons. We found that there was no minimum level of thermal conductivity as the period length changed. The thermal conductivity was always lower than that of the uniform 3C/4H-SiC nanowires. The reduction in the thermal conductivity may be attributed to the lack of periodicity and increased disorder along the 3C/4H-SiC nanowires.

**Figure 6:**
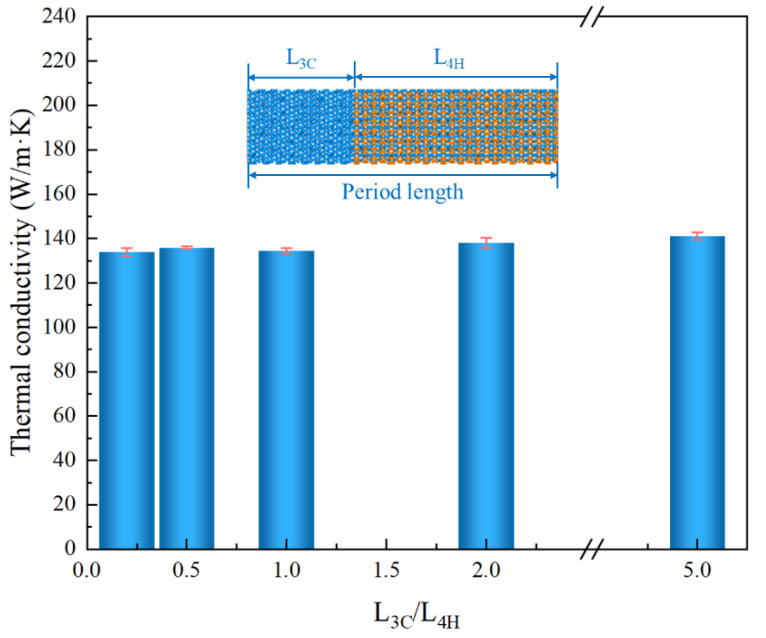
The dependence of thermal conductivity on the ratio of 3C/4H for 3C/4H-SiC nanowires. The uniform period length was 179.31 Å.

With a fixed uniform period length of 179.31 Å, the effect of the ratio of 3C/4H length on the thermal conductivity was investigated, as shown in Figure 6. The insert in Figure 6 shows the structure of 3C/4H-SiC when the ratio of 3C/4H length was 1/2; the lengths of 3C and 4H were 59.77 Å and 119.54 Å, respectively. The thermal conductivity was shown to weakly depend on the ratio of 3C/4H length. Compared to the simulation model with a ratio of 3C/4H = 1, the thermal conductivity change rate was 2.71% and 5.03% with the 3C/4H ratio of 2 and 5, respectively. In contrast, when the ratio was less than 1, only slight fluctuations in thermal conductivity occurred; the thermal conductivity change rate was 0.28% and 1.13% with the 3C/4H ratio of 0.2 and 0.5, respectively.

### 3.4. Effect of Temperature

**Figure 7:**
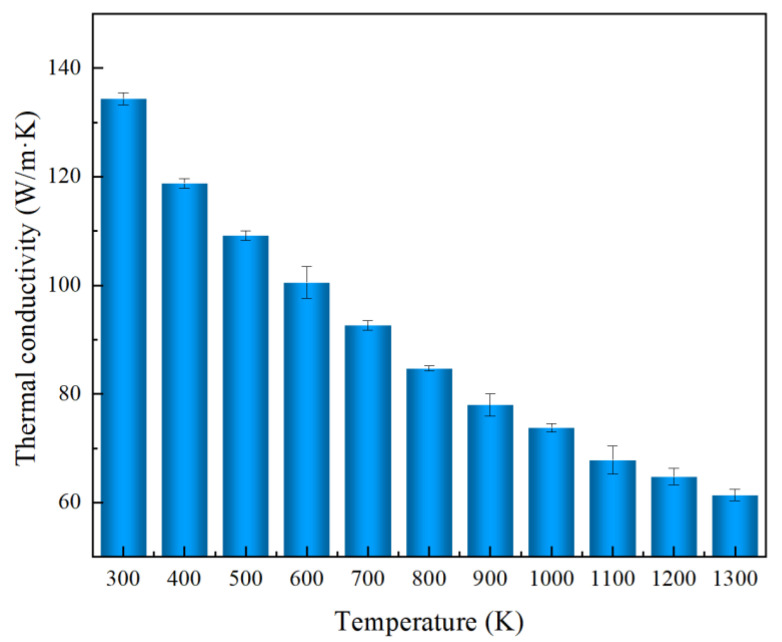
The temperature dependence of thermal conductivity on the 3C/4H-SiC nanowires. The uniform period length was 179.40 Å.

As we all know, bulk SiC materials have good thermal stability at high temperatures. However, the thermal stability at high temperatures is reduced when the dimensions are reduced to the nanoscale [65]. We performed relaxation calculations for 3C/4H-SiC at different temperatures. Results showed that the structure of 3C/4H-SiC can be maintained stable at temperatures below or equal to 1300 K. Thus, the thermal conductivity of 3C/4H-SiC nanowires with a uniform period length at temperatures from 300 K to 1300 K was investigated, and the results are shown in Figure 7. The sample total length and uniform period length were 717.60 Å and 179.40 Å, respectively. We found that the change in the thermal conductivity with temperature exhibited the typical behavior of crystalline solids. The thermal conductivity of the uniform period 3C/4H-SiC nanowires decreased as the temperature increased, which we attributed to the increasing phonon–phonon Umklapp scattering [66].

### 3.5. Effect of Strain

**Figure 8:**
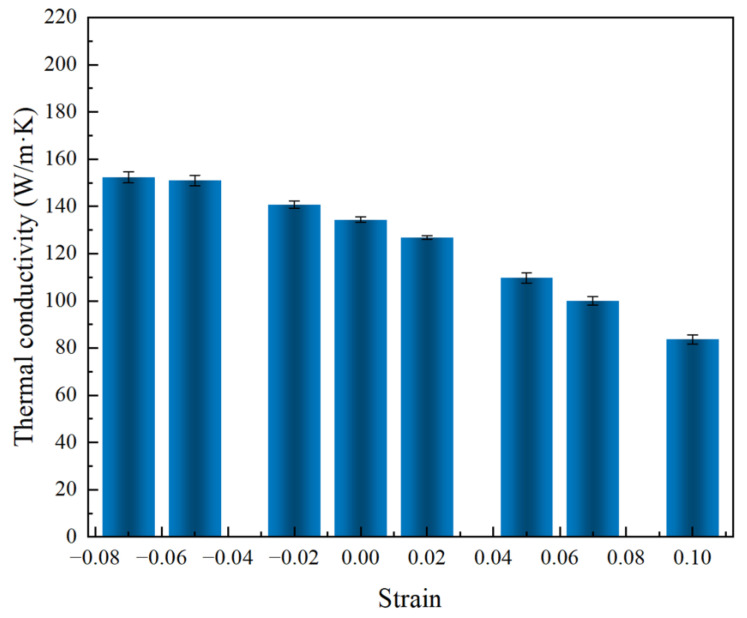
Strain effect on the thermal conductivity values of the 3C/4H-SiC nanowires at 300 K. The uniform period length was 179.40 Å.

Various situations, such as externally applied mechanical loading, lattice mismatch at epitaxy interfaces, thermal expansion, etc., have been reported to generate strain. The resultant change in thermal conductivity can be remarkable. The effects of uniaxial strain parallel to the thermal transport direction (along the *z* axis) on the thermal conductivity of the 3C/4H-SiC nanowires at 300 K were investigated and are shown in Figure 8. The strain was defined as $\varepsilon =(l\;-l_{0})/l_{0}$, where $l_{0}$ and l are the length of the simulation box in the strain loading direction for unstrained and strained samples, respectively. The results showed that the thermal conductivity of the 3C/4H-SiC nanowires decreased continuously from compressive strain to tensile strain. The thermal conductivity increased along with the compression strain applied to the sample. When the compression strain was -0.07, the thermal conductivity increased by 13.37%. The thermal conductivity decreased with the tensile strain applied to the simulation sample. When the tensile strain was 0.07, the thermal conductivity decreased by 25.6%. This phenomenon of decreasing thermal conductivity from compressive strain to tensile strain can be attributed to uniaxial strain-altering phonon behavior [67]. When 3C/4H-SiC nanowires are subjected to compressive strain, phonon dispersion curves shift towards high frequencies, resulting in greater phonon group velocity of the acoustic phonons. Such a shift in the phonon dispersion curve will result in an increase in the specific heat. Under tensile strain, the trend follows the low-frequency direction, and both phonon group velocity and specific heat decrease [68,69]. Thus, the thermal conductivity decreases continuously when the compressive strain changes to the tensile strain.

## 4. Conclusions

To summarize, the NEMD simulation was employed to investigate the thermal conductivity of 3C/4H-SiC nanowires. Our results reveal that the Tersoff-1990 potential can accurately describe the acoustic phonon branches along the high-symmetry point of the Brillouin zone. The thermal conductivity of the 3C/4H-SiC nanowires was significantly lower than that of the perfect monocrystalline 3C- and 4H-SiC nanowires due to the interfacial thermal resistance. The thermal conductivity demonstrated nonmonotonic variation with the uniform period length. The existence of a minimum level of thermal conductivity (134.36 ± 1.16 W/m·K) as a function of uniform period length could be attributed to the coherent-to-incoherent transition. The thermal conductivity of the uniform period nanowires reduced with the increasing temperature due to enhanced phonon–phonon Umklapp scattering. The thermal conductivity monotonically decreased with strain from uniaxial compression to tension by changing the phonon group velocity and specific heat. In addition, the thermal conductivities of uniform period nanowires exhibited irregular fluctuations with the ratio of 3C/4H length. As for the gradient period of 3C/4H SiC nanowires, their thermal conductivity weakly depended on the gradient period lengths due to the lack of periodicity and increased disorder. Our investigation provides a feasible method by which to reduce the thermal conductivity of SiC, thereby promoting its application in thermoelectricity.

## Figures and Tables

**Table 1:** Fabrication of SiC materials.

Raw Material	Fabrication Method	Reference
SiC powder, rice husk	Spark plasma sintering (SPS)	[26,27,28,29]
SiC powder, SiC whiskers	Hot isostatic pressing (HIP)	[30,31]
SiC fiber, polycarbosilane	Cold isostatic pressing (CIP)	[32,33]
4H–SiC substrate	Liquid-phase epitaxy (LPE)	[34]
SiC powder, Si powder, C powder	Physical vapor transport (PVT)	[35,36]
Silicon wafers, methyltrichlorosilane	Chemical vapor deposition (CVD)	[16,37]

## Data Availability

The data that support the findings of this study are available from the corresponding author upon reasonable request.
